# Decisions that hasten death: double effect and the experiences of physicians in Australia

**DOI:** 10.1186/1472-6939-15-26

**Published:** 2014-03-25

**Authors:** Steven A Trankle

**Affiliations:** 1 Centre for Health Research, School of Medicine, University of Western Sydney, Campbelltown, Australia

**Keywords:** Euthanasia, Assisted-suicide, Principle of double effect, Force majeure, End-of-life care, Qualitative research

## Abstract

**Background:**

In Australian end-of-life care, practicing euthanasia or physician-assisted suicide is illegal. Despite this, death hastening practices are common across medical settings. Practices can be clandestine or overt but in many instances physicians are forced to seek protection behind ambiguous medico-legal imperatives such as the Principle of Double Effect. Moreover, the way they conceptualise and experience such practices is inconsistent. To complement the available statistical data, the purpose of this study was to understand the reasoning behind how and why physicians in Australia will hasten death.

**Method:**

A qualitative investigation was focused on palliative and critical/acute settings. A thematic analysis was conducted on semi-structured in-depth interviews with 13 specialist physicians. Attention was given to eliciting meanings and experiences in Australian end-of-life care.

**Results:**

Highlighting the importance of a multidimensional approach, physicians negotiated multiple influences when death was regarded as hastened. The way they understood and experienced end-of-life care practices were affected by politico-religious and cultural influences, medico-legal imperatives, and personal values and beliefs. Interpersonal and intrapsychic aspects further emphasised the emotional and psychological investment physicians have with patients and others. In most cases death occurred as a result of treating suffering, and sometimes to fulfil the wishes of patients and others who requested death. Experience was especially subject to the efficacy with which physicians negotiated complex but context-specific situations, and was reflective of how they considered a good death. Although many were compelled to draw on the Principle of Double Effect, every physician reported its inadequacy as a medico-legal guideline.

**Conclusions:**

The Principle of Double Effect, as a simplistic and generalised guideline, was identified as a convenient mechanism to protect physicians who inadvertently or intentionally hastened death. But its narrow focus on the physician’s *intent* illuminated how easily it may be manipulated, thus impairing transparency and a physician’s capacity for honesty. It is suggested the concept of “force majeure” be examined for its applicability in Australian medical end-of-life law where, consistent with a multidimensional and complex world, a physician’s motivations can also be understood in terms of the emotional and psychological pressures they face in situations that hasten death.

## Background

In the world of end-of-life care, Australia holds a position of political and cultural importance ever since legalising the world’s first medicalised euthanasia program - The Rights of the Terminally Ill Act [1,2]. For a brief period between 1996 and 1997 Australia’s Northern Territory enacted legislation that would allow dying patients, upon meeting specific criteria, to gain medical assistance and control the timing and manner of their death [3]. Since then, other countries have legalised euthanasia, for example, the Netherlands and Belgium in 2002 [4] and Luxembourg in 2008 [5] while Switzerland has provided legalised physician-assisted suicide since 1941 which it also extends to non-nationals [4,6]. In the USA, physician-assisted suicide was legalised in Oregon in 1997 [7], Washington State in 2008 [8] and Montana in 2009 [9,10]. However, as it is elsewhere, in Australia such death hastening practices remain illegal despite overwhelming public support [11-13], and consistently strong support from physicians and nurses [14,15]. Indeed, new pro-euthanasia/assisted suicide bills are continually presented before various Australian State legislatures. To date, all have been defeated by varying margins through conscience votes, although voting is often still along party lines [16]. Accordingly, physicians who intentionally or inadvertently hasten death in the course of providing end-of-life care cannot draw legislative and, thereby, clear legal support.

However, death hastening practices, frequently at a clandestine level, continue unabated across Australian medical settings and by a multidisciplinary range of practitioners [17,18]. Such practices sometimes occur without patient consent (up to half the cases) and sometimes with nurses and others acting autonomously without instruction from doctors [14,19]. Indeed, over one third (36.5%) of all Australian deaths are caused or hastened by medical end-of-life decisions but over two thirds are subject to them [15,20,21].

Often the way euthanasia or assisted suicide is conceptualised by individual physicians is inconsistent [11,22-24] and particular practices like increasing analgesia, treatment withdrawal, and divisive interventions such as sedation [25,26], all of which may hasten death, can only be considered from the context in which they are administered. For example, in jurisdictions like the Netherlands, euthanasia is understood to be performed at the explicit voluntary request of patients and through pharmacological administration. However, death hastening practices more broadly can also be performed without the request of patients and through withdrawing life sustaining measures.

In a large multinational survey, Löfmark et al. [27] highlighted the ambiguity in how physicians understand death hastening practices. Of 1,478 Australian physicians, 7% reported complying with patient requests for euthanasia with 28% willing to comply under certain conditions and around 66% stating they would never comply. However, 77% reported withholding or withdrawing treatment while 83% intensified the alleviation of pain through analgesia with the probability or certainty of hastening death, and some conceptualised these practices as euthanasia. Accordingly, it appears that many Australian physicians in this study may have performed euthanasia at least once, yet on the request of patients very few complied (7%). This discrepancy further suggests physicians are performing these practices without patient consent.

Moreover, although those opposed to euthanasia state that good palliative care renders it unnecessary [28], research with 1,100 GPs working in end-of-life contexts in New Zealand, reported 693 (63%) had made medical decisions in the previous 12 months that could actually hasten death [29]. Thirty-nine (5.6%) deaths were consistent with physician-assisted suicide or euthanasia. In 17 of these deaths doctors did not discuss their actions with the patient. In other words, non-voluntary euthanasia occurred, and in 34 (87%) of the 39 deaths palliative services were available. The authors strongly suggested that doctors did not consider palliative care adequate in meeting patient needs and supports findings using the same questionnaire in Australia, Belgium and the Netherlands [21,30,31]. Ninety-four (13.6%) New Zealand physicians reported actions that were “partly” intended to hasten death and 50 (53%) of these did not discuss with the patient beforehand. Furthermore, 132 (19%) withdrew or withheld treatment or increased medications to alleviate symptoms knowing it would probably hasten death.

Physicians often confront end-of-life situations that require specific decision making. In order to provide appropriate end-of-life care that considers the dying patient, patient loved ones and collegial multidisciplinarity, physicians need to negotiate a multitude of influential factors. This is consistent with a multidimensional understanding of end-of-life care where interactive influences at macro, meso and micro levels affect how physicians conceptualise, negotiate and experience the care they provide [32-35]. For example, macro influences like politics, religion and media; meso influences such as medico-legal prescriptions and profession-specific ideologies (e.g. comfort vs. cure) are brought to bear with the micro level influences of physicians such as their own personal beliefs, and moral/ethical positions. Physicians further engage with multicultural influences that include diversity in individual beliefs and attitudes of patients, loved ones and professional colleagues to make sense of practices.

Such complex influences are important considerations when seeking to understand the ambiguity and inconsistency in physicians’ end-of-life care beliefs and positions, and the practices they engage in. Furthermore, their efficacy in negotiating multiple influences at the bedside shapes their experiences [36]. Indeed, physicians experience burnout from personal [37-39] and also interrelated organisational influences [40,41], and research further identifies increasing numbers of physicians in Australia leaving their profession due to unsustainable demands being placed on them [42-45].

A complex multidimensional world renders singular and less inclusive considerations explanatorily inadequate. Yet medico-legally, physicians need to operate under uni-dimensional imperatives if patient deaths are potentially hastened. For example, the Principle of Double Effect (PDE) has long been a protective mechanism that physicians draw on in such situations and where they may attract professional or legal retribution [46,47]. However, the PDE provides generalised rather than situation-specific guidelines that more often only considers the *intent* of the physician [48]. Specifically, a physician is culpable if the intent is to kill but not if the intent is to alleviate suffering even with a foreseeable outcome of death ensuing as a direct consequence. Although the PDE recognises proportionality in comparing good and bad outcomes, it does not consider other extenuating influences in the physician’s motivations such as unique moral or emotional factors that may be relevant. Indeed, compassion may be a particularly strong motivator. Further, if a physician intends to end life, practices can easily be reframed as intended to address intractable suffering. The PDE is acknowledged as problematic [48] and remains controversial despite efforts to improve and reinvent the guideline [49,50].

Few jurisdictions other than the Netherlands recognise the complexity in end-of-life decision making where patient requirements, and the physician’s interaction with them, represent a unique dynamic. Prior to legalising euthanasia and assisted suicide there in 2002, a defence of *force majeure* could be drawn on by physicians to better elucidate their intent and culpability before the law in cases where death was hastened [51,52]. A physician’s actions could be understood medico-legally as ascribing to a “superior force”, where they were morally and professionally compelled to address the suffering of patients and as subject to the emotional and psychological pressures inherent in end-of-life decision making [53,54]. Such considerations, however, are not available to physicians in Australia.

The aim of this report is to document accounts of physicians in Australian palliative and critical/acute settings who need to negotiate death hastening practices in the context of providing end-of-life care to dying patients and patient loved ones. Accordingly, the utility of current medico-legal imperatives is examined. Physicians’ experiences and ability to assist “good deaths” are considered in relation to multiple contextualised influences.

## Method

This report draws from a larger body of qualitative research investigating end-of-life care, the purpose of which was to bolster limited knowledge, particularly in the Australian context. The ambiguity and inconsistency in physicians’ practices and experiences, identified in an extensive literature review [55], was examined by addressing the primary research question: *How do physicians understand, negotiate and experience end-of-life care decision-making and practices in the context of Australian critical/acute and palliative settings?* A supportive research question considered: *What influences a physician to affect the timing of death?* Interview questions centred on personal experiences and individual beliefs and positions, good and bad deaths, and elicited information on practices where death might be hastened (Appendix). However, a semi-structured approach allowed a great deal of physician direction and thus the potential for revealing other interesting paths of inquiry. In-depth interviews were one-on-one with the author and each between 60–90 minutes duration.

Prior to embarking on this research, approval was obtained from the University’s Human Research and Ethics Committee (# H7589). Letters of introduction, a research information sheet, and an informed consent document were generated for distribution to potential participants. A purposive recruitment strategy provided participants with relevant experience in end-of-life care, and across different clinical settings, with around half recruited through a snowball strategy after referral from their colleagues. Direct contact was made by the author to16 physicians; 13 responded and agreed to participate. One was known personally to the author, some were selected through their professional register and others because of their published research. Sampling comprised seven palliative care specialists, three intensive care specialists, one respiratory/thoracic specialist and two GPs. Participants were between 36 and 68 years of age, their experience in providing end-of-life care varied from six to over 40 years, and eight physicians were male. Most practiced from hospitals and hospices across the Sydney metropolitan area, two were from regional areas and two were from another capital city. Due to the sensitive and possibly illegal and professionally damaging nature of the data physicians provided, they were guaranteed confidentiality and anonymity. Each was provided with a pseudo-name, third party information was de-identified, and all interviews were transcribed exclusively by the author.

A thematic analysis, through an inductive/data driven approach, was conducted according to guidelines established by Braun and Clarke [56]. Thematic analysis was compatible with the multidimensional approach and theoretical considerations of the research [34], and particularly useful for examining data that was previously unexplored. Correspondingly, the interpretive nature of analysis generated future research questions and directions.

As part of a larger body of research, trustworthiness was ensured by triangulation, which occurred through discussing the coding frame with my advisory panel and arguing its rationale according to the data, and investigative approach driving the study. Further, and assisted through NVivo, an extensive series of notes and memos were generated to facilitate a process of checking and re-checking. These began by recording observational data and thoughts upon leaving interviews, and then throughout the analysis and written report. To ensure analytical rigor, deviant or discrepant cases were also evaluated. Data collection concluded when themes became saturated and nothing new emerged. It is also important to acknowledge that some researcher influence is unavoidable in any research design and particularly in qualitative research where interview dynamics have a large impact on the data obtained. Being aware of my reflexivity and documenting this, allowed me to attend systematically to every step of the research process. Finally, thematic and analytical integrity was assessed through successive chapter drafts leading to a completed thesis, and reviewed by panel members who approved for examination. The following analysis presents and discusses selected key themes.

## Results and discussion

The first two key themes, and their subthemes, concentrate on how physicians understand and position themselves in relation to practices that hasten death. Meaning is articulated. The remaining themes focus on their corresponding experiences and recognise how the physician’s fundamental beliefs and positions, and how they negotiate end-of-life situations, influence experiential aspects.

### Religion and sanctity of life

Religion and its concomitant sanctity of life position [57,58] provide support to physicians who hold the belief that death should not be deliberately hastened. Control over life and death (and its timing) is with nature or the divine [59]. Accordingly, such a position precludes any acceptance of a request to die or any intentional autonomous action of the physician to hasten death. Although religiosity is characteristic of hospice and palliative settings, and a position held by nearly all palliative specialists, it also flows through broader political, legal and professional positions, and physicians *across* specialties held similar views.

To illustrate, Aaron (Respiratory/Thoracic Specialist) who works in critical/acute settings does not support euthanasia in end-of-life care: *“I’d have a problem with active euthanasia or mercy killing, but from my religious standpoint and my religious beliefs in my medical practice, I obviously don’t practice it”.* He identifies a religious structure that influences his views (and medical practices) but suggests euthanasia, while also describing it as merciful, is still an ambiguous issue for him: *“I could see the rare situation where it would be appropriate but I don’t know if I would ever become involved in that”.* He acknowledges that intentionally hastening death could be appropriate in some cases which highlight the unique nature of individual deaths and how each must be considered in its context.

Similarly, as a practicing Christian, Jeremy (Palliative Specialist) identifies the influence religion is for him: *“I have involvement in the clergy and that dimension in terms of training and expectations. That’s not what I’m employed for…but that does obviously colour my views and attitudes and what I bring to this field”.* Accordingly, he also fundamentally opposes intentionally hastening death. He holds the sanctity of life position which regards euthanasia as deliberately inducing death rather than alleviating suffering:

I do feel personally very strongly…that doctors are doctors and not executioners, and if you want to legalise euthanasia then you appoint executioners. Please leave the medical profession out of it, and I don’t see euthanasia as the ultimate relief of suffering. I see that as inducing death deliberately.

Jeremy strongly defines the role (and identity) of doctors as medical practitioners and not executioners. Death should not be exclusively under their control: *“I think that life is valuable and it’s not for us to decide the time for our birth or our death”.* There is sanctity in life, and death as with birth is not a medical decision; he suggests life and death are subject to nature or the divine. Like Jeremy, Kerrie (Palliative Specialist) also holds the sanctity of life position and, consistent with medico/legal doctrine, rejects any support for an intentionally hastened death:

I don’t believe we should terminate life, I think it’s going to end for all of us…I think we need to be very respectful of life, I can’t change the outcome but I can continue to care. I think these are very vulnerable people, and I think if we set precedents, then there’s lots of other vulnerable people in society where we’re effectively saying “Ok your life is rubbish, let’s end this”.

Kerrie mentions being “respectful of life”, it is not for her to terminate. She accepts the inevitability of death but how she can still care. She identifies those dying as “very vulnerable people” and fears a precedent if death is hastened. Kerrie clearly draws on the slippery slope argument [60]. Similarly, and although patient suffering is a prime consideration for Gary (Palliative Specialist), it does not override his fundamental belief that control over the timing of death should not rest solely with him:

I can certainly understand their suffering and their family’s suffering, but… it’s just, an area I couldn’t go down, even if euthanasia became legalised, I’m not sure that’s something I could do…even if I knew it was relieving their physical suffering. I think it’s such a minefield, you know, who you do this for…are you trying to end someone’s life because of the suffering of the family or…the patient…

Gary empathises with patient (and family) suffering but illustrates how the euthanasia issue is difficult for him. He identifies the slippery slope argument by questioning who he is actually providing euthanasia for, and that if legalised he would still have difficulty performing it. Around half of the physicians in the Netherlands who deal with requests to hasten death have reported similar reluctance to comply [61]. But Gary’s religious and family influences also emerge here: *“it might be the religious upbringing in me that says it’s wrong to end someone’s life and I suppose, my own family, what’s right and wrong…my father was a policeman who….was very black and white”.* Gary acknowledges multiple social influences of family, religion and law as shaping his views on hastening death and on what he considers right and wrong.

The multiplicity of interactive influences seen in Gary’s excerpts signify complex relationships in the environment between nested macro, meso and micro elements. For example, religion and the sanctity of life position are embodied at a cultural and political level, enforced at a legal and professional level, and influences Gary’s family and his upbringing. He internalises those influences which subsequently shape how he regards end-of-life care. The influential power of religion and the sanctity of life position for controlling the timing of death, and subsequently influencing personal and professional beliefs, were similarly seen in the excerpts from Aaron, Kerrie and Jeremy.

#### Patient choice and autonomy

Contrary views to such commonly accepted positions on hastening death are, however, also provided by some physicians who consider patient choice and autonomy most important in directing their position on controlling the timing of death. Indeed, Kohlberg’s highest stage of moral reasoning described abstract universal ethical principles valid for all humanity regardless of concrete laws and social agreement [62]. Right action is defined by self-chosen ethical principles of conscience [63]. In the following excerpts, physicians further described their personal ethics by not only positioning themselves as a patient and being guided by what they would like in that position, but also a personal belief that control over death should be in the hands of the individual. For example, Peter (General Practitioner) illustrates his empathy for others: *“I always think what would I want if this was me”?* He places himself in the patient’s position, conveying a desire to be able to direct end-of-life care. Peter believes patients should have a choice to elect death if that is their wish, but in making the distinction between euthanasia and physician-assisted suicide, he encourages patient agency: *“I think in principle I’d feel more comfortable that the person who’s acting is clearly acting of their own volition. You’re providing them with the drugs but they are taking the drugs, there’s something nice about that”.* Peter indicates his preference to assist rather than control patient deaths. He considers death as “something nice” when it can be achieved through patient choice and volition.

Correspondingly, Robert (Palliative Specialist) further illustrates the intrapsychic of physicians, specifically the personal influences that could motivate a physician to intentionally hasten death: *“I think if ‘patient autonomy’…was your guiding ethical principle…and your guiding treatment motivation was ‘compassion’…and, ‘mercy’…then, they would be two ethical and motivating factors…that could lead you down the road of helping those people, which are…I think, positive ethical principles”.* Robert talks of personal ethical principles in terms of autonomy, compassion and mercy and these reflect his philosophy of promoting patient choices and helping people. Differing somewhat from his palliative colleagues, Robert does not rule out hastening patient deaths. However, holding such ethical principles might not be enough to accede to a request for hastened death:

It’s not just respect for autonomy, and compassion and mercy, but it’s also a certain amount of courage and willingness to take risk…and, all of those things would need to line up…and, then of course there’s the means to do it as well and having access to that…which isn’t always…which could be a huge challenge.

Robert additionally identifies courage, willingness and risk to hasten death. Certainly one needs to be willing to do something that is contrary to the beliefs of many others and might not always sit well with one’s own beliefs, but particularly courage to take the risk of social and professional ostracism and legal consequences. He also talks about access and means. It might be difficult performing a hastened death because of the suspicion it might arouse when patients are also monitored by others, where medication charts record patient dosages and pharmaceuticals are also securely stored and accounted for. Multiple structures might need to line up, for example, personal aspects, opportunity within institutional frameworks, professional collaboration and a means of protecting oneself professionally and legally such as double effect. Otherwise, as Magnusson has identified in the Australian context, attempts to hasten death may be unsuccessful [17].

#### Motivated by suffering

Further identifying the importance of personal beliefs, some physicians who might reject the sanctity of life position or religious prescriptions will consider patient suffering as the determinant of whether death is hastened or not. Most will still stay within accepted professional guidelines, while others consider these of lesser importance compared to the suffering of patients and the need to do whatever is required. For example, when Peter (General Practitioner) is confronted by patient suffering he regards control over death as a matter of choice and necessity, and not something under natural, divine or legal control. *“Because I don’t really respect the ethic ‘not use deliberate means to hasten death’, I see no conflict in these instances and see them as being entirely compatible really…because relieving suffering sometimes involves hastening death”.* Peter’s personal views on hastening death are not constrained by normative beliefs that preclude intent. Similarly, although not advocating the intent that Peter does, Gina-Leanne (Intensive Care Specialist) is also motivated by patient suffering and pain. She does not consider inadvertently hastening death problematic within the critical/acute setting she works in and its focus on saving lives:

If they’re obviously suffering…whether that’s with, pain…or whatever it is, and you know for certain that they are going to die and whatever it is that they’ve got is not amenable to anything…it’s not going to make a lot of difference really whether they die now or in 48 hours’ time, but I guess my aim is to make them comfortable and if in the process of making them comfortable that hastens their death well so be it.

Time appears relative in hastening death. In the course of a lifetime, 48 hours is insignificant for Gina-Leanne. Particularly with intractable suffering and a prognosis of imminent death, Gina-Leanne’s aim is to comfort even if medication plays a role in the dying process. She is clearly drawing upon double effect.

### The active/passive distinction and double effect

Although many physicians overtly support the sanctity of life position on hastening death, when death is inadvertently (or otherwise) hastened, the active/passive distinction [47,64] is drawn upon by some physicians to conceptualise and account for their actions. Some consider a hastened death due to administration of narcotics differently to a death hastened by the withdrawal of life sustaining treatment. Similarly, the principle of double effect provides another way of understanding and rationalising physician actions. Legal/professional imperatives influence the position some physicians hold on hastening death.

Many physicians state that they would not intentionally hasten death regardless of whether it is requested or not, but death sometimes occurs as a consequence of addressing patient suffering. This is something Thomas (General Practitioner) said: *“look my goal is not to kill anyone but if I need to get on top of their suffering and they die, then that’s ok”.* Sometimes physicians draw on the active/passive distinction when death is hastened foreseeably or otherwise, and Jenny (Palliative Specialist) illustrates this distinction: *“not giving a life prolonging treatment is different to…uhm giving the medication, which inadvertently might hasten death, so I think they’re different”* Although outcomes might be the same, Jenny understands a more qualitative difference between withholding versus providing treatment that may hasten death. Furthermore, she also uses the word “inadvertently”, which suggests double effect. Indeed, like Thomas above, most physicians in this study referred to end-of-life practices where their explicit intent was to alleviate suffering even though their patient sometimes died during that process.

However, Keith (Intensive Care Specialist) draws on double effect while making the active/passive distinction to negotiate a patient request to hasten death:

I say, basically, “look I can understand where you’re coming from but it’s not legal in this country to actively end life…but having said that, what you’re really saying is you don’t want this treatment escalated…and I’ll understand that and I’ll withdraw anything that we’re doing and make sure you’re not suffering”, and usually they’re happy with that.

Keith finds the distinction useful to somewhat accede to a request but protect himself legally and professionally. He emphasises the word “actively” when ending life, but it is almost a subversive approach where Keith needs to reframe the meaning of a potential intervention along legal and professional lines. He provides patients with a safer option that addresses suffering and hastens death. Some level of sedation or increased analgesia is suggested when Keith “makes sure the patient is not suffering”, but he illustrates how death hastening interventions can be manipulated by reframing their meaning relative to intent. Indeed, the notion of intent is slippery and Peter (General Practitioner) describes sedation as an effective vehicle to disguise intent:

I mean it was slow. It has to be slow; it goes under the name of “slow euthanasia”. I said: “well how long it takes is really a legal issue. If I do it slowly I’m very safe”. If I come along and give you a massive morphine injection and say “here that’ll fix your pain” I can’t defend it. So I’ve got to be able to defend myself.

Peter provides a common understanding of sedation as “slow euthanasia” [65], but he clarifies for a patient how hastening her death through sedation needed to be slow if it was to afford him legal and professional protection. He identifies the ambiguous distinction between a legal action that may hasten death (inadvertently) and one that is more overt but illegal.

### Feeling emotional pressure: The influence of others requesting death

Most physicians experienced emotional pressure from patients and their loved ones requesting a hastened death. Often they would *beg* or *plead* with the physician to be compassionate and bring about a hastened death to end suffering. To illustrate, Robert (Palliative Specialist) talks of emotional pressure applied by patients to hasten their death: *“it’s much harder legally to…ah accede to a patient’s…you know, request…but it’s more than a request, it’s not a demand because they can’t demand uhm…but it’s a plea really”.* Robert identifies requests more as a “plea”, where patients and others place themselves at the mercy of the physician but also bring significant emotional pressure to bear. Acknowledging the legal framework he must work within, Robert mentions the difficulty of acceding to a request for death, but it may also be “harder” for Robert because the decision comes with added emotional pressure. Gary (Palliative Specialist) discussed this also: *“you know some patients and their families are literally begging you to end it…it’s really tough sometimes but you can understand their suffering”.* Gary empathises with patient and family suffering and it is “tough” for him when they “beg” for death. Similarly, Candice (Palliative Specialist) said: *“you feel the sadness, you empathise…some are in such despair and plead with you to be compassionate and end their burden”.* Although palliative specialists who were generally focused toward a comfort-based approach to care experienced emotional pressure to hasten death, physicians who were more curatively oriented reported frequent experiences of this nature. End-of-life settings are not the same and intensive care is not a nice place to die. Patients receive invasive procedures and therefore requests to die are common. Often they are unable to speak, particularly when intubated, and provide intensive care specialists like Keith with handwritten notes: *“I’ve got several…bits of paper with people writing ‘please let me die’…in shaky handwriting…many people don’t want to go on when they’re in intensive care, tubes, lines and painful procedures, and strangers…”*.

#### Dogs die a better death: Requests as control over vicarious suffering

Certainly, many dying patients request to die but Jeremy (Palliative Specialist) also points out that requests to hasten death are frequently made by family members of the dying patient. They consider that their loved one is needlessly suffering a protracted dying process, but suffering is also implicit for those who endure vicariously at the bedside:

It’s not that uncommon, where families say things like: “look…if Dad was the family dog, you’d deal with it”, you know…“it shouldn’t happen to a human being; we treat our family pets better”. Those sorts of things get said, or you know, “how long is this going to go on for”?

Jeremy describes the common analogy many family members make of treating the family dog better. Families appeal to the compassion in their physician to be humane and end the suffering of their loved one. Kerrie (Palliative Specialist) also describes the analogy Jeremy provides, where patient loved ones say: *‘“come on Doc, this is awful’, ‘Doc you wouldn’t leave your dog like this’, yeah ‘I’m a country person; we don’t let our animals suffer like this”’.* When families’ make requests to hasten the death of their loved one, sometimes it is to also address their own suffering. Indeed, Maggie (Palliative Specialist) believes that most requests come from families, rather than patients, and suggests they are often made on that basis: *“the majority of direct requests come from families, who are just struggling sitting in the room and watching…the physical process of dying”.*

### A hastened death can be a good death

Physicians are not only involved in a professional relationship with dying patients and patient families; empathy and rapport also indicate that relationships carry personal and intrapsychic components. Physicians are affected when patients die and by how they die. Insomuch as the literature identified physicians leaving the profession and experiencing burnout, and disengagement, it is important to cite the positive aspects of hastening death because such experiences may counter those more negative and assist physicians to remain motivated and engaged, and continue providing exceptional end-of-life care while maintaining their own health and well-being. Bearing witness to intolerable suffering might often be an aversive experience, particularly when treatment interventions are limited. Yet, physicians have measures at their disposal which can address such situations and culminate in a positive experience. Physicians report that sometimes a good death is a hastened one.

Indeed, Gary (Palliative Specialist) is really glad he can sedate to end a patient’s extraordinary suffering:

There are times when I’m really glad I can use sedation…I remember a guy about 10 years ago with mesothelioma…who I went to see one morning and his eyes were literally bulging, he was gripping the bedside table and really struggling with every muscle trying to breath, so, I mean you can’t do anything else in this situation, because there was nothing reversible, and ah…he was going to stay like that. And it was through his breathlessness he pleaded with me to do something…so I told him what I was going to do, and consulted the family…said ok…this is what we’re going to do, and once we start this over the next four hours he will get sleepy and he’s not going to wake up.

Gary highlights how he collaborated with the patient and his family on the treatment decision and provided them with clear information on what the outcome would be. Clearly, Gary knew that the consequence of the treatment was death. But he had little choice to address the level of suffering he was confronted with. The patient was literally *begging* him to do something, placing Gary under considerable psychological and emotional pressure. He remembers this well even after 10 years. Yet Gary considers the outcome positively. He “felt good” turning a difficult situation into a “good death”: *“I’m glad I got to him when I did, because nobody should suffer like that. Within a few minutes he settled…and a few hours later passed away peacefully. I felt good…it ended up a good death considering his situation”.* Earlier Gary declared he opposes deliberately hastening death, but when confronted by intractable suffering, he shows he will do whatever he needs to for the patient, even if it shortens life. Similarly, Aaron (Respiratory/Thoracic Specialist) is motivated to end suffering regardless of whether it hastens death: *“look, I don’t believe in hastening death for just any reason. But there have been patients suffering so much that there’s little else I could do but treat it…and sometimes they died…but I’m happy with that…I’ve stopped the suffering”.* Aaron feels “happy” hastening the deaths of patients because they no longer suffer. Like Gary, he has limited options to do otherwise.

Keith (Intensive Care Specialist) further illustrates how death hastening practices may be manipulated with sedation to satisfy legal and professional imperatives, and provide a satisfying outcome that addresses extraordinary suffering.

You know I feel really lucky, because I’m in a position to stop suffering…there was a fellow drowning in his own…lung water because his heart was failing so badly, he was really suffering, so I put him to sleep, put a tube down him just to stop all of the secretions coming out, but I sedated him so much I had to take over his breathing otherwise it would have killed him, and I said “we’re just going to put you off to sleep now” knowing that they were the last words he’d ever hear…and then I sort of weaned him off the ventilator…he was deeply unconscious and 10 seconds later I pull the tube out and now I’m withdrawing treatment because it’s futile, but there’s only a minute between me giving the big dose of sedation and him actually dying…but you see, why did I intubate and then extubate? I did it because I didn’t want it to look like euthanasia…but that’s pretty close to euthanasia isn’t it?…I just couldn’t watch that suffering…nobody can watch a man drown, I mean in his own blood and sputum, he was in so much distress…but that’s pretty close isn’t it?

Keith indicates his intention to address what seems like extraordinary suffering for this patient in the best way he could, by ending life. However, he illustrates how highly procedural it needs to be to bring about an outcome that also protects him legally and professionally, where *intent* is assisted by the active/passive distinction. He regards the action almost in terms of euthanasia when he says “that’s pretty close to euthanasia isn’t it”. But he goes further into an almost involuntary euthanasia position when he declares “knowing they were the last words he’d ever hear” with the patient told he was only going off to sleep. This might have avoided further patient distress but it might also have been Keith acquiescing to an earlier request by the patient. Keith’s motivations to address suffering are quite clear when he talks about nobody being able to watch that amount of suffering. This begs the question of whose suffering he was trying to alleviate. Keith stated when he can pro-actively address such suffering: *“it’s a very satisfying experience, whereas 20 years ago I wouldn’t have thought that was satisfying. I wouldn’t have even thought about it perhaps”.* Keith tends to think about these instances more than he once did, but recounts this experience as “very satisfying”. These excerpts identify the importance of context, where specific dynamics play out that uniquely influence in the moment. For example, Keith’s experience is subject to an interaction with the patient, his developed attitudes, and professional/legal considerations.

But some physicians, who are not in a position to intervene directly, educate their patients in the terminology of double effect. For example, Peter (General Practitioner) deals with requests to die and supports patients who seek his advice: *“I’m completely sympathetic to it”.* As their GP he acts in a somewhat consulting role and advises patients on how they might obtain a hastened death from their *treating* physician:

I’ve been involved with a lot of people who have been dying difficult deaths with cancer…where as their GP I’ve suggested, that the best way in this situation to get what they want is to try the following strategy with their doctors…and ah…that’s sometimes been helpful in the sense that they’ve been able to talk their doctors into taking them through one of these uhm…double effect deaths…I feel good helping patients like these who have nowhere else to go.

Confronted by many patients suffering difficult deaths, Peter enjoys helping them by providing information and strategies on how they might approach their treating physician and encourage them to assist hastening their death. Asking their physician for a “double effect death” has been helpful to patients. The treating physician is protected legally and professionally, and the patient’s wish to die is accommodated. Peter suggests that some physicians might more readily consider a request to die if it is made by a patient informed with a practical strategy. Legal considerations are a necessary priority for Peter when he advises patients to seek a double effect death, and he shows how medico/legal constraints can be negotiated, but there are also times when he steps over that legal threshold:

Uhm…on occasions I’ve talked to them about how they ah, and I’ve got to be a little bit careful here, because this is illegal, uhm…but I’m aware of the fact that if they were to take certain drugs that they’re being prescribed, in certain ways, that they would get the peaceful death that they want, so ah, and those situations are always coming up, and certainly I haven’t got the slightest moral qualm about my…involvement, I feel quite pleased about it. I think it was the right thing to do, but it isn’t the lawful thing to do. And…it’s one thing suggesting that they talk to their doctors about double effect…but it’s another thing entirely to suggest that they take this particular drug that they’re being prescribed by another source, in a certain way. And that’s obviously illegal.

Peter goes beyond double effect, and provides information to patients on how misusing the medication that another physician has prescribed can bring about the outcome they are seeking. Such situations are frequent and he certainly acknowledges the illegality of giving such advice, but, in complete support for his patients, he declares he does not have the slightest moral qualm. Peter makes his position clear indicating, that although illegal, “it was the right thing to do”; he identifies a personal moral and ethical framework that guides him. He also regards his involvement as “quite pleasing”. This is understandable when he can practice consistent with his beliefs, and also successfully negotiate medico/legal constraints.

### Double effect is an ambiguous scaffold

However, hastening death is often not experienced so positively by physicians. They are required to negotiate limited treatment options within a constraining system of care. Most regard double effect as inadequate and fundamentally flawed but sometimes it is their only protective medico/legal option. Double effect is a generalised guideline, yet each situation and opportunity is unique and sometimes physicians have restricted capacity for agency within institutionalised structures. They cannot always practice as they consider most appropriate. At such times they can experience their provision of care aversively. For example, Aaron (Respiratory/Thoracic Specialist) says: *“it’s really difficult when some patients suffer intractably and nothing you give works. Sedation isn’t perfect but sometimes it’s all you have…you can’t euthanase even if that seems the most appropriate thing”.* Similarly, Keith (Intensive Care Specialist) says: *“I know some patients suffer terribly…it’s tough watching that but there isn’t always opportunity to give them what they want and so I manage them as best as I can”.*

Like a number of his colleagues, Peter (General Practitioner) described the lack of options, of patients being trapped within institutional structures, where performing a hastened death at a patient’s request was fraught with difficulty. However, the system is sometimes negotiated to bring about a hastened death, albeit within a very limited range of options. Although successfully accomplishing a hastened death requested by his patient, he described the *required process* as “disgusting” and “obscene”:

I described it as pretty…obscene, I mean she woke up at one stage there, after some 24 hours in what is it, in almost an induced coma that we were administering using increasing levels of narcotics and Midazolam…she woke up at one stage when the infusion stopped, in the middle of the night…and asked her best friend who was a nurse who was helping us with the whole process…because what we were trying to do was bring about her death, ah…“Am I dead”? It was just bizarre that…“Katy am I dead”? I remember it, because I was asleep in the next room, and there’s this scream from Katy who comes in and wakes me up and said she’s woken up the drip’s stopped. They got the drip started then she lapsed back into unconsciousness again and, it’s an obscenity, I mean we’re not seeing good medicine here, we’re seeing an obscene practice under the guise of medical process and ah nothing, nothing I suppose…I think it’s a, pretty disgusting situation, now that doesn’t mean that I think that doctors who take this path…should be criticised, it’s the only option that the current legislation allows them, I mean no one’s suggesting doctors should go and throw themselves on some sacrificial pile, if they want to help you, and they have to do it in a way that they stay safe themselves, this really is the only option.

Peter is describing a death brought about by sedation, a legal practice *manipulated* to achieve an outcome suitable for the patient. He highlights the problematic (and potentially traumatic) nature of such a divisive intervention with the patient waking up during the process of her death. Again supporting his medical colleagues, Peter levels blame for such obscene deaths at the medical *system*, which constrains options for hastening death. He acknowledges legal and professional concerns of physicians who elect to take this path without legislative support.

Sometimes physicians are confronted by extraordinary suffering, where actively administering narcotics is the only remedial choice but dramatically shortens life. Such situations often endure psychologically for the physician who struggles to reconcile outcomes with their personal or professional positions on hastening death. For example, Gary (Palliative Specialist) said: *“when you treat intractable suffering like that and the patient dies…sometimes you struggle…you go over it and over it in your head and ask yourself if there was another way”.* Similarly, Jeremy (Palliative Specialist) describes such a case where he hastened death, and experiences ongoing self-recrimination:

Now that’s not to say that I haven’t killed people. I remember very clearly…a well-known figure, who was in the terminal stages of a terminal illness…extraordinarily distressed with unrelieved pain and breathing difficulties and thrashing around on the bed, and nothing that had been given was working. And I drew up a syringe full of Midazolam, and I popped it into a vein, and I just pushed it in and pushed it in and pushed it in until his breathing settled down and he relaxed, and he stopped thrashing around, and found ah, an induced peace if you like. Now a quarter of an hour later he died. I cannot tell you whether my intention was solely to relieve his suffering, or to address my suffering, or to address the suffering of the staff and others who knew this man. I can’t unpack that in all honesty…I know that in all probability the medication I gave him did hasten his death…I still struggle to unpack that for myself.

This was an incident that occurred over 20 years ago but still troubles Jeremy. He questions whose suffering he was trying to alleviate and struggles to unpack and come to terms with the experience. Physicians like Jeremy find themselves in a difficult position. They mustn’t hasten death according to religious doctrines (which he and others like Gary and Aaron strongly support) or professional and legal requirements, yet they cannot witness extraordinary suffering and do nothing either. They have an obligation to their patient*.*

Physicians, who sometimes negotiate hastened deaths, often regard being compelled to find protection within ambiguous legal frameworks as inadequate and inappropriate. Double effect is divisive within the medical community [48,66]; indeed Jenny (Palliative Specialist) said: *“the old term ‘double effect’ shouldn’t be used anymore…I think if opioids are used appropriately it should never happen; that’s the reason why palliative care is so extremely important in Australian end-of-life care and worldwide end-of-life care”.* She promotes the value of her specialty and the expertise of its practitioners, denying that double effect is still required. Certainly, some physicians like Peter and Keith above will actively manipulate practices to hasten a death and subsequently draw on double effect; but others will only use it reluctantly or of necessity. For example, when his intractably suffering patient died shortly after being treated, Jeremy (Palliative Specialist) acknowledged how he could be compelled to use double effect despite finding it distasteful:

I don’t like invoking the Principle of Double Effect as justification for what I did with that patient…at all. But, uhm…,…I know that I could. I know if someone attacks me in law I would have to use that argument to say that, that I was not responsible for his death…but I feel responsible.

Using double effect does not sit well with Jeremy. He has no other option, however, if confronted with prosecution. Although he might find legal and professional protection, the situation remains difficult for him. He acknowledges the protection he has but also identifies how double effect does not assuage his sense of responsibility for a patient’s death. This is something he is left to deal with.

Furthermore, Robert (Palliative Specialist) identifies how the law only considers the doctor’s “intent” when a death is deemed to be hastened*.* He suggests patients implore their physician to be compassionate and assist a hastened death. There is significant emotional and psychological pressure brought to bear on the physician by the patient and also by the system or context the physician is immersed in. Robert suggests that there is little capacity in the law to recognise the motivations of physicians when a death is hastened. Nonetheless, the physician is forced to negotiate the needs of patients within a legal and professional framework but in a way that is also consistent with their own position on particular practices:

Not just a petty crime, the worst of all crimes, all hinging on this concept of intention which, I think, is where there should be some reform, to the ethical and legal framework, because…the person’s autonomy, what they’re looking to the doctor to do, the doctor’s motive of compassion and understanding and professional responsibility to help people who are suffering and struggling…all of those things are morally relevant and they should be legally relevant…I shouldn’t be made to feel like a criminal if I’m helping my patient!

Robert talks about other issues beyond a simplistic evaluation of intent that are also important moral considerations in a hastened death. He mentions compassion, understanding, a respect for patient autonomy and a responsibility to address suffering. Legal imperatives should not subordinate moral relevance. He proposes a review of current narrow and simplistic laws:

Yeah the law hinges on just one thing: was it in the doctor’s mind to intend bringing about this guy’s death? And…that’s a very simplistic and crude way of framing it, and why I think there should be a review of that law. It’s really hard focusing on the patient when I have to always worry about what might trip me up. And I do worry about that.

He explains how the focus he needs to keep on his patient is distracted by “always worrying” about potential legal ramifications for the practices he engages in. But there is little or no recognition in double effect of the emotional or moral pressure physicians are placed under by patients and others to accede to a hastened death.

### Limitations

Although this study provided unique insights into Australian end-of-life care practices and the experiences of physicians when death was regarded as hastened, some research limitations merit consideration. Sampling was particularly appropriate for this qualitative investigation, albeit with a potential self-selection bias. Of note, many physicians reported some positive experiences around hastening death, but *all* of them overwhelmingly reported death hastening experiences as distinctly negative. This suggests that such experiences are more widespread among physicians elsewhere. Accordingly, a mixed method approach utilising characteristics of the present study, could elicit both quantitative and qualitative data from a greater number of physicians and enhance the current findings. Additionally, the present study accessed practicing physicians, yet many others with similar experiences who have ceased practicing for any multitude of reasons, some of which may include burnout, could also provide valuable data. It is also important to acknowledge interview dynamics, with meaning and knowledge being “located” and a partly co-constructed effort, where no two interviews (even if repeated with the same interviewee) could ever be the same. Consistent with a multidimensional and complex world, there is more than one truth [32]. It is for this reason that a more generalised research approach suggested above could complement rather than diminish the present research.

## Conclusions

This report has illuminated how the Principle of Double Effect (PDE) is a divisive and inadequate medico-legal structure. Nonetheless, the PDE is frequently drawn on by physicians across Australian settings in situations where death is regarded as hastened. Physicians are compelled to negotiate the end-of-life requirements of patients and patient loved ones from within a constraining legal framework, the outcomes of which influence the experiences of all who are involved.

Situated within its larger study, this report further addresses the paucity of qualitative research examining end-of-life care in the Australian context. Although statistical data has informed the prevalence of physician practices that hasten death, there is now a greater understanding of why and how physicians engage in such practices. Through a multileveled approach, the complexity of influences behind hastened deaths and how physicians make sense of and experience them could be more inclusively elucidated. Indeed, the corresponding ambiguity and inconsistency reported in the literature [55,65] is better understood when considering context specificity, which influences how end-of-life decisions may be negotiated, and illustrates how the positions and beliefs of physicians might not always be compatible with practices.

A multidimensional and complex world recognises the importance of political and religious doctrines, and medico-legal imperatives, and acknowledges the uniquely personal of the physician as directive in how care is administered and subsequently experienced. Physician experiences and well-being are important considerations for the continuation and development of exemplary end-of-life care. Legal guidance, that considers more than only the intent of physicians in difficult decisions that may end life, could be one way of ensuring physician motivation, continued engagement at the bedside and the capacity for good deaths.

Indeed, it may be worthwhile revisiting the Netherlands and how the concept of *force majeure* was applied before euthanasia became legal. Future policy research may examine cases where force majeure was drawn upon and upheld, and how such a concept may be applied to the Australian context. Corporate law in Australia recognises force majeure where “acts of God” are considered a superior force. In a similar way, physicians deciding on practices that may hasten death are also subject to superior forces which are of a psychological, emotional and moral nature.

Until death hastening practices are considered not only under criminal law but also medical law by legalising euthanasia, force majeure may be an option to create greater transparency and acknowledge the physician’s actions in terms of emotional, psychological and moral pressure. Manipulation of double effect might be minimised yet each case where death is hastened could submit to individual scrutiny and assessment before the law if required. A narrow concept of “intent” has long been acknowledged as problematic but nonetheless is the legal imperative physicians are *still* required to operate under [46-48].

As explicitly stated by some physicians in this study, and implied by many others, a review of current medico-legal guidelines is indicated, especially if we consider it important that physicians should be able to act out of compassion to a much larger extent. Legal acknowledgment of the multifactorial influences reflected by unique bedside dynamics may better support physicians and the interests of their patients, and more often culminate in good deaths. A physician’s capacity to assist good deaths is also contingent upon maintaining their own emotional and psychological well-being, and practicing consistent with their personal ethics and beliefs where patients’ needs are not necessarily subordinated by a general legal need, is one way of achieving this.

## Appendix

### Interview Schedule

Research Question: *How do physicians understand, negotiate and experience end-of-life care decision-making and practices in the context of Australian critical/acute and palliative settings?*

Could you please tell me a little about your role in end-of-life (EOL) care?

– What is your background?

– What EOL settings do you have experience with (e.g. GP, palliative, critical, oncology, gerontology)?

– How long have you been providing EOL care in Australia?

Could I ask you, what do you see as a “good death”?

– Can you give me an example from your experience when a patient had a “good death”?

– What happened?

– What was that like for you? *(Explore deeper and seek a potential contrary)*

– What did you think about that?

– How did you feel about it?

– When are you not able to assist a good death? (*Setting/other constraints?)*

– Why?

– An example?

What do you see as a “bad death”?

– Could you describe an example from your experience in which a patient had a “bad death”?

– What happened?

– What was that like for you? *(Explore deeper)*

– What did you think about that?

– How did you feel about that?

There may be times in end of life care when an intervention to alleviate suffering has the foreseeable but unintended consequence of hastening death. *(Increased analgesia-sometimes non-titrated due to restlessness; withholding antibiotics for pneumonia, withdrawing nutrition/hydration in PS etc.)*

– Could you give me an example from your experience when this occurred?

– What happened (in terms of a good or bad death)?

– What was that like for you?

– What were your thoughts about that at the time?

– How did you feel on that occasion?

– What other experiences have you had when using such end-of-life interventions?

– How have those experiences been different?

OR

– When would you consider such interventions appropriate?

– What has been your experience with patients making a “specific” request for you to alleviate their *suffering* through death-hastening means?

– Could you describe a time when you received a *patient request* for assisted death?

– What happened?

– What did you do?

– What was that like for you?

– What were your thoughts about that?

– How did you feel on that occasion?

Research shows that suffering is often much more than physical, for example existential suffering, anxiety or fear over the progression of illness, loss of functional integrity and dignity, and loss of autonomy and independence, have been linked more strongly than physical pain to patient requests for hastened death.

– What has been your experience in receiving *patient requests(for death)* to end that kind of suffering?

– What happened?

– What did you do?

– What was that like for you?

– What were your thoughts about that?

– How did you feel on that occasion?

Have you had some experience with dying patients who, perhaps considering themselves a burden on others, felt it was their “duty to die”?

– Could you describe a time when you received a request to hasten death from a patient who considered it their “duty to die”?

– What happened?

– What was your response?

– How did you feel receiving such a request?

– Could you describe a time when you received a *request from the patient’s family or loved ones* to hasten a suffering patient’s death?

– What happened?

– What did you do?

– What was that like for you?

– What were your thoughts about that?

– How did you feel on that occasion?

Could you describe a situation where you considered making or would consider a decision to shorten the life of a dying patient who was suffering intractably?

– Could you provide an example?

– What was that like for you?

– What were your thoughts about that?

– How did you feel on that occasion?

When would you consider it appropriate (in terms of “good” or “bad” deaths) to “intentionally” hasten a patient’s death?

– What would it take?

– Are there any differences or exceptions?

Are there times when you experience a conflict between your ethical and professional duty to relieve suffering and your ethical and professional duty not to use means which deliberately hasten death?

– Could you describe a time when you had such a conflict?

– What happened? What did you do?

– How did you feel?

Are there times where you experience a conflict between your own personal standards and beliefs your ethical and professional duty to relieve suffering without hastening death?

– Could you describe a time when you had such a conflict?

– What happened? What did you do?

– How did you feel?

– Could I just ask you a couple of final questions?

– Firstly, what’s the best part of your job?

– and not really wishing to end on a bad note, but what’s the worst part?

(End of interview)

Is there anything we might have missed or that came up in the interview that you would like to cover or talk further about?

### Supplemental interview questions

(If not raised by the physician, used selectively and when appropriate).

Could I just ask you about your experiences with terminal or palliative sedation as an end of life practice?

– Could you give me an example where you used sedation with a particular patient?

– What happened in terms of a good or bad death?

– How did you feel on that occasion?

– Is sedation a valid or practical surrogate or alternative to VE or PAS?

– Why or why not?

Patient care for those with a terminal prognosis ultimately needs to progress from a critical or acute focus to one more palliative, for example, the cessation of more aggressive forms of treatment to a regime that emphasises comfort and palliation.

– Could you describe how you negotiate new care goals and treatment options with patients and their loved ones as they become necessary for patients with irreversible disease?

– What are some of the problems you encounter in such negotiations?

– Could you give me an example of a situation when you needed to do this?

– What happened?

– How did you feel?

## Competing interests

The author declares he has no competing interests.

## Author’s information

Steven Trankle recently graduated from his PhD Candidature with the Centre for Health Research in the School of Medicine at the University of Western Sydney. His thesis was titled “End of Life Decisions and Practices: The Experiences of Doctors in Australia” [55].

## Pre-publication history

The pre-publication history for this paper can be accessed here:

http://www.biomedcentral.com/1472-6939/15/26/prepub
